# Supplementary figures and images for: CRISPR Screens Reveal Epstein-Barr Virus-activated JunB as a Key Lymphoblastoid B cell Dependency Factor that Represses Cyclin Dependent Kinase Inhibitor P18INK4c
Source: bioRxiv. 2026 Apr 1:2026.03.31.715635. Preprint. [Version 1] doi: 10.64898/2026.03.31.715635 (PMC13060307; doi:10.64898/2026.03.31.715635)

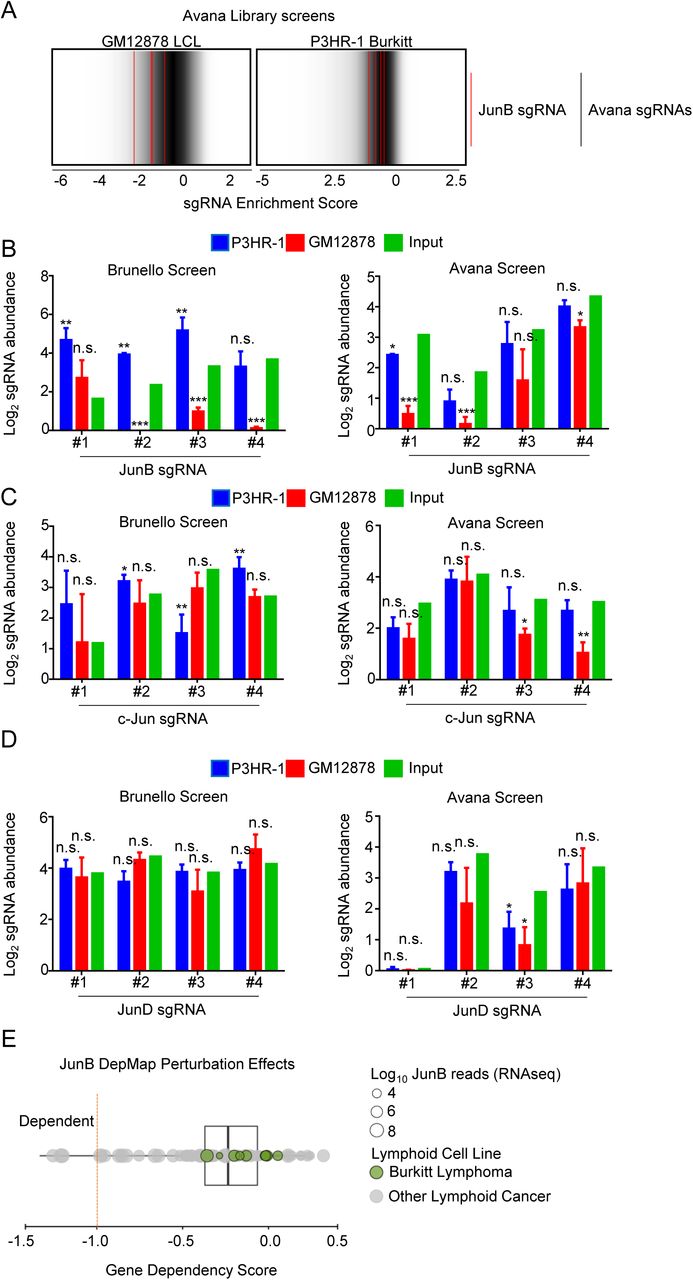

Supplement: figure-S1 — Supplementary Figure 1: JunB knockout affects lymphoblastoid cell growth. (A) Log2(fold change) of the four guides targeting JunB (red) compared to the distribution of all sgRNA guides from the GM12878 and P3HR-1 Avana dropout screen. Fold change was calculated comparing abundance of sgRNA day 21 post selection versus input. (B) JunB sgRNA abundances in Brunello and Avana dropout screens. Log2 JunB sgRNA abundances from GM12878 (red) and P3HR-1 (blue) cells 21 days post library selection were determined via deep sequencing and compared to input library sgRNA abundance (green). (C) c-Jun sgRNA abundances in Brunello and Avana dropout screens. Log2 c-Jun sgRNA abundances from GM12878 (red) and P3HR-1 (blue) cells 21 days post library selection were determined via deep sequencing and compared to input library sgRNA abundance (green). (D) JunD sgRNA abundances in Brunello and Avana dropout screens. Log2 JunD sgRNA abundances from GM12878 (red) and P3HR-1 (blue) cells 21 days post library selection were determined via deep sequencing and compared to input library sgRNA abundance (green). (E) JunB dependency across CRISPR DepMap lymphoid cell lines (DepMap.org). Gene dependency scores were calculated by comparing JunB sgRNA abundance in individual lymphoid cell lines 21 days post lentivirus library transduction and selection in comparison to input library abundance. Each circle indicates an individual lymphoid cell line. Circle size represents Log JunB reads from RNA-seq analysis (DepMap, Broad, DepMap Public 25Q3 (2025)) Green circles versus clear circles indicate DepMap Burkitt versus non-Burkitt lymphoid cell lines, respectively. P-values were determined by one-sided Fisher’s exact test. * p<0.05, **p<0.005, ***p<0.0005 [file figure-S1.jpg]

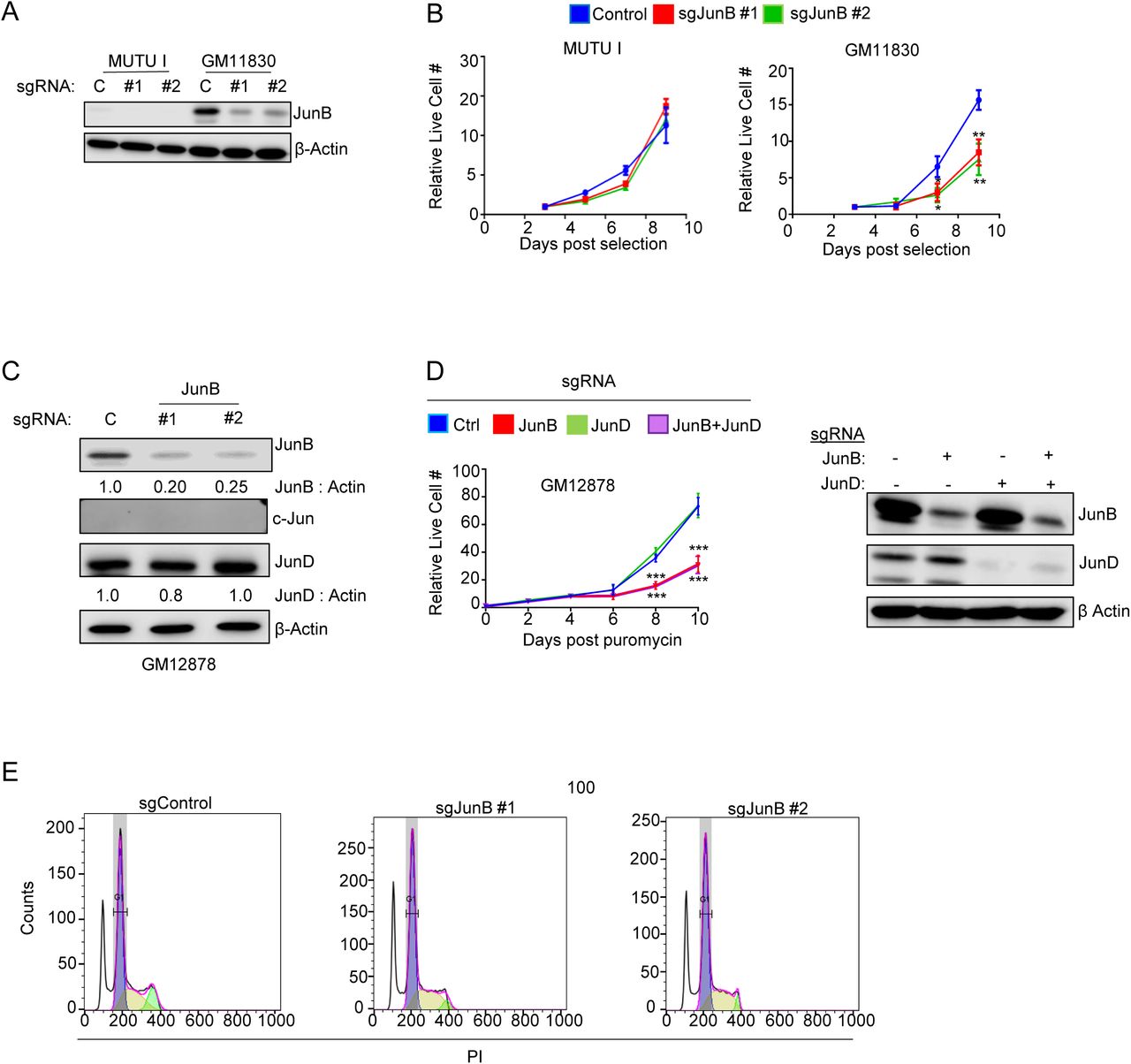

Supplement: figure-S2 — Supplementary Figure 2: JunB, but not c-Jun or JunD, regulates EBV transformed cell growth and cell cycle (A) Validation of JunB CRISPR-Cas9 knockout in MUTU I and GM15892 cells. Immunoblot was performed on WCL from Cas9+ MUTU I and GM15892 cells transduced with control or JunB sgRNA seven days post-selection using indicated antibodies. (B) Mean ± SD live cell numbers of Cas9+ MUTU I or GM11830 cells expressing control or JunB targeting single guide RNAs (sgRNA) from n=3 replicates. Cells transduced with lentiviruses expressing the indicated sgRNAs were puromycin selected. Cell numbers two days following puromycin selection (defined as day 0 of the graph) were set to 1. Live cell numbers were quantitated by CellTiter-Glo assay (C) Investigation of impact of JunB on other Jun AP-1 transcription factors. Immunoblot analysis of WCL from Cas9+ GM12878 cells transduced with control or JunB sgRNAs, ten days post selection, with indicated antibodies. Densitometry ratios of JunB and JunD to β-Actin is shown. (D) Investigation of combinatorial impact of JunB and JunD on LCL growth. (Left) Mean ± SD live cell numbers of Cas9+ GM12878 cells expressing control, JunD, JunB, or JunB+JunD targeting single guide RNAs (sgRNA) from n=3 replicates. Cells transduced with lentiviruses expressing control or JunD sgRNA were Zeocin selected two days post transduction. After one week, cells were then transduced with a separate control or JunB sgRNA. Cell numbers two days following puromycin selection (defined as day 0 of the graph) were set to 1. Live cell numbers were quantitated by CellTiter-Glo assay. (Right) Immunoblot validation of JunB and JunD knockouts. WCL of GM12878 cells transduced with both zeocin and puromycin sgRNAs was extracted two days following puromycin selection and immunoblot was performed using indicate antibodies. (E) PI cell cycle stainin of GM12878 cells transduced with JunB sgRNA. Representative FACS cell cycle plots from n=3 replicates of GM12878 cells analyzed te [file figure-S2.jpg]

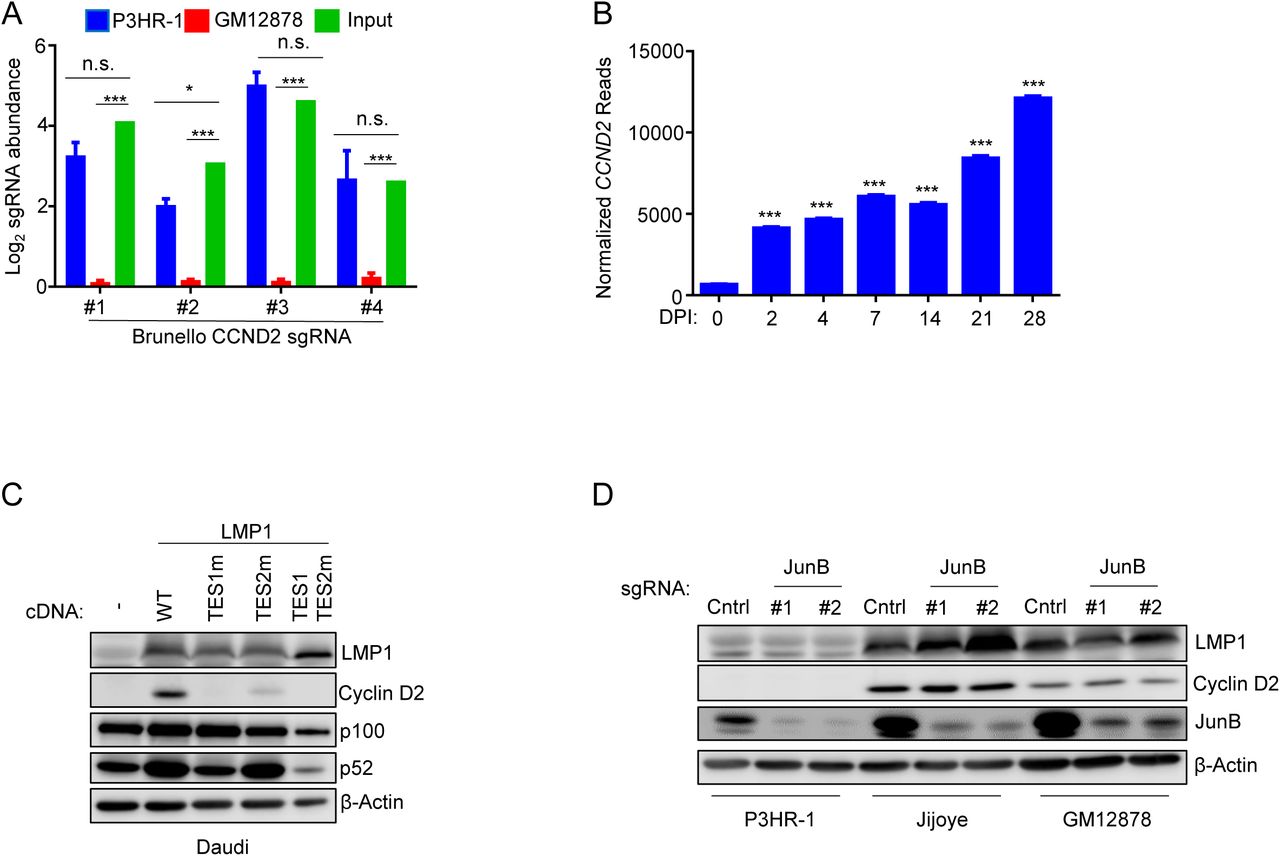

Supplement: figure-S3 — Supplementary Figure 3: Epstein-Barr virus Latent Membrane Protein 1 regulates CCND2 levels, but not via JunB. (A) CCND2 sgRNA abundances in Brunello P3HR-1 and GM12878 dropout screens. Log2 CCND2 sgRNA abundances from GM12878 (red) and P3HR-1 (blue) cells 21 days post library selection were determined via deep sequencing and compared to input library sgRNA abundance (green). (B) CCND2 transcript levels during EBV-mediated B cell transformation. RNAseq was performed on primary B cells infected with B95-8 virus on indicated days post infection (dpi). Normalized reads for CCND2 are shown. (C) Immunoblot analysis of CCND2 levels in response to LMP1 expression. Daudi Burkitt cells harboring selectively inducible WT or transformation effector site (TES) mutant LMP1 cassettes were mock induced or treated with 250 ng / mL doxycycline for 24 hours. Cells were then harvested and lysates analyzed via immunoblot with indicated antibodies. (D) Effect of JunB knockout on CCND2 levels. Cas9+ P3HR-1, Jijoye, or GM12878 cells expressing control or JunB sgRNAs. Seven days post selection, WCL was extracted from cells and immunoblot performed using indicated antibodies. P-values were determined by one-sided Fisher’s exact test. * p<0.05, **p<0.005, ***p<0.0005 [file figure-S3.jpg]
